# A spiking neural network model of 3D perception for event-based neuromorphic stereo vision systems

**DOI:** 10.1038/srep40703

**Published:** 2017-01-12

**Authors:** Marc Osswald, Sio-Hoi Ieng, Ryad Benosman, Giacomo Indiveri

**Affiliations:** 1 Institute of Neuroinformatics, University of Zurich and ETH Zurich, Zurich, Switzerland; 2 Université Pierre et Marie Curie, Institut de la Vision, Paris, France

## Abstract

Stereo vision is an important feature that enables machine vision systems to perceive their environment in 3D. While machine vision has spawned a variety of software algorithms to solve the stereo-correspondence problem, their implementation and integration in small, fast, and efficient hardware vision systems remains a difficult challenge. Recent advances made in neuromorphic engineering offer a possible solution to this problem, with the use of a new class of event-based vision sensors and neural processing devices inspired by the organizing principles of the brain. Here we propose a radically novel model that solves the stereo-correspondence problem with a spiking neural network that can be directly implemented with massively parallel, compact, low-latency and low-power neuromorphic engineering devices. We validate the model with experimental results, highlighting features that are in agreement with both computational neuroscience stereo vision theories and experimental findings. We demonstrate its features with a prototype neuromorphic hardware system and provide testable predictions on the role of spike-based representations and temporal dynamics in biological stereo vision processing systems.

Depth perception is an extremely important feature for both natural and artificial visual processing systems. It is an essential requirement for many practical applications, ranging from fine object manipulation in robotics, to driving in autonomous vehicles. One of the most common techniques employed by both living beings and machines to achieve depth perception is based on *stereo vision*. However this process is subject to the well known *stereo correspondence problem*, which deals with the challenge of finding visual correspondences of the same features from two different views. Finding these correspondences in natural scenes is a complex and error prone process. Erroneous matches of visual features (false targets) lead to the perception of wrong depths. The correct depth of a visual scene can be successfully inferred only if the correspondence of true targets is established. While animals, including even insects^1^ solve this problem effortlessly and efficiently, current machine vision systems are struggling with the efficient implementation of their underlying complex algorithms. The strong demand of today’s machines to perceive their environment in 3D has recently driven the field of machine vision to become a very active area of research in stereo vision^2^,^3^. The solutions proposed however require significant computational resources, which have a strong effect on power consumption, latency, throughput and the system’s physical size, making it difficult to integrate them on compact devices.

Here we present a novel approach to the stereo correspondence problem, inspired by biological stereo vision systems, which is compatible with ultra low latency and low power neuromorphic hardware technologies^4^. In particular, we exploit advances made in both mixed signal analog/digital VLSI technology and computational neuroscience which enabled us to combine a new class of retina-like artificial vision sensors with brain-inspired spiking neural processing devices to build sophisticated real-time event-based visual processing systems^5^,^6^,^7^. Rather than capturing static frames and transmitting sequences of frames discretized in time, the neuromorphic vision sensors we use transmit continuous streams of spikes, or “address-events”^8^,^9^, which are produced by the individual pixels when the brightness contrast they sense changes by an amount greater than a set threshold. All of the pixels in the sensor are independent and send their address asynchronously, in continuous time, on a digital bus, as soon as they generate a spike. Similarly, the neuromorphic processors we recently developed^10^,^11^ use address-events to receive, process, and transmit spikes. Input address events represent spikes that are delivered to destination synapse circuits, and output address-events represent spikes produced by the source silicon neurons. These neuromorphic processors typically comprise large arrays of silicon neurons that can be arbitrarily configured to implement different types of neural networks, for processing real-time event-based data such as data produced by silicon retinas^10^.

The availability of these event-based neuromorphic sensors has led to an increased interest in studying and developing a new class of event-based vision algorithms^12^,^13^,^14^,^15^,^16^,^17^. However, most of these algorithms have been used in conjunction with standard computing architectures, rather than neuromorphic processors, and to a large extent are still biased by the frame-based approach typically adopted in classical machine vision. In this work we follow a drastically different approach that moves away from the classical computer science mindset: we propose an event-based model of stereo vision that unifies the domains of perceptual neuroscience and machine vision, and that can be implemented end-to-end using spike-based neuromorphic technologies. The model consists of a spiking neural network capable of computing stereo correspondence from the visual stream of neuromorphic vision sensors. The network has been designed using computational primitives that are available in existing neuromorphic VLSI processors. Given the dynamic properties of the neural networks implemented by these processors, their co-localization of memory and computation, and their extremely compact sizes^4^, they represent a possible solution to the von Neumann bottleneck problem^18^ which enables stereo vision systems built in this way to be integrated in compact low-power systems. Furthermore, as our model is well connected to established models of stereopsis, its implementation based on neurons and synapses that exhibit biologically realistic time constants suggests a potentially important role for precise temporal dynamics in biological stereo vision systems.

## Results

The main results presented in this Section originate from numerical simulations of the stereo network performed on a standard desktop computer. The spike-based visual data used as input to the network was either obtained through real recordings made with neuromorphic silicon retinas or was synthesized in artificially, depending on the type of experiment performed. This section however also demonstrates the validation of the numerical simulations performed using a full custom neuromorphic VLSI device, for a proof of concept demonstration.

### The spiking stereo neural network

**Figure 1:**
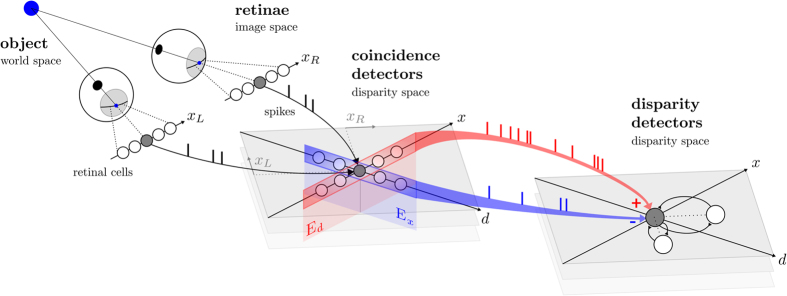
The spiking stereo network. Detailed view of a horizontal layer of the network. An object is sensed by two eyes and accordingly projected onto their retinal cells. The spiking output of these cells is spatio-temporally correlated (coincidence detectors) and integrated (disparity detectors). The final output encodes a representation of the original scene in disparity space (*x, y, d*). For the sake of visibility, only a horizontal line of retinal cells, at fixed vertical cyclopean position *y*, is considered. The corresponding coincidence and disparity detector units, hence, lie within a horizontal plane (spanned by *x* and *d*). Only a few units are shown here whereas in the complete network, the units are uniformly distributed over the entire plane. The shaded planes indicate how the network expands vertically over *y*. More details on how the neurons are connected among each other is provided in the Methods section.

The spiking neural network we propose is inspired by the well established cooperative network of Marr and Poggio^19^, but is characterized by two major differences: first, the input to the network does not consist of static images but of dynamic spatio-temporal visual information in the form of spike trains which are directly obtained from event-based neuromorphic vision sensors (see the Methods section); second, the network is composed of Integrate-and-Fire spiking neurons operating in a massively parallel fashion, which are self-timed and express temporal dynamics analogous to those of their real biological counterparts. The retinal cells are represented by two populations of ON and OFF neurons which serve as the input to the network. These cells project with excitatory connections to a second population of neurons that act as “coincidence detectors”. A third population of neurons, termed the “disparity detectors”, pools responses from the coincidence detector neurons using both excitatory and inhibitory connections. To improve the detection of correct correspondences while suppressing disparity responses to false targets, the disparity neurons implement a winner-takes-all mechanism via recurrent inhibitory connections. A detailed view of a horizontal layer of the network is illustrated in Fig. 1. Each spike from a retinal cell represents a change in illumination at a specific spatial position at a particular time. For each pair of corresponding horizontal lines of retinal cells, a horizontal layer of coincidence neurons signals temporally coinciding spikes. These cells are arranged such that each one has a unique spatial representation in disparity space (*x, y, d*) (only *x* and *d* are shown) such that each spike provides evidence for a potential target at the corresponding real-world disparity position. Disparity space represents a relative map of the three-dimensional world with the *disparity* coordinate *d* = *x*_*r*_ − *x*_*L*_ and the *cyclopean horizonal* and *vertical* coordinates *x* = *x*_*R*_ + *x*_*L*_ and *y* = *y*_*L*_ = *y*_*R*_ respectively (a detailed explanation of the coordinate system and the map can be found in the Methods section). The population of coincidence detectors therefore encodes all potential targets (including true and false disparities). The disparity detectors implement a binocular correlation mechanism, which is realized by integrating the responses from coincidence detectors within the plane of constant disparity *E*_*d*_, which is spanned by (*x, y*) and the plane of constant cyclopean position *E*_*x*_, which is spanned by (*d, y*). Activity in *E*_*d*_ constitutes supporting evidence for true correspondences (excitation of disparity detector), whereas activity in *E*_*x*_ denotes countervailing evidence (inhibition of disparity detector). Finally, a uniqueness constraint is enforced by mutual inhibition of disparity detectors that represent spatial locations in the same line of sight.

### How the stereo network solves the correspondence problem

The disparity detector neurons compute an approximation of the covariance of the interocular visual input (see Supplementary Material). Although these detectors show selectivity to true targets, they also respond to false targets when similar features are present. The inhibitory interactions among disparity neurons reduce this effect, but do not completely resolve ambiguities because the relatively large receptive fields of the disparity detectors smooth-out the disparity response. To resolve these ambiguities we consider the spikes generated by both the coincidence and the disparity detectors: the network produces a “disparity event” only when the event produced by a disparity neuron is coincident (i.e. happens within a few milliseconds) with a spike produced by a coincidence detection neuron at an equal (or nearby) representation in disparity space (see Supplementary Material for details).

**Figure 2:**
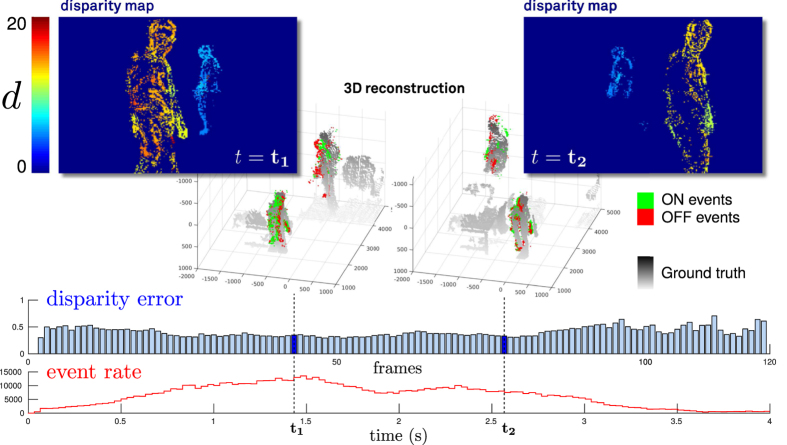
Successful resolution of the stereo correspondence problem by the spiking neural network. The recorded scene consists of two people that move in opposite directions at different depths. Here, the two depth maps were generated by binning the output spikes of the network into 30 ms bins at times *t*_1_ and *t*_2_ respectively. The corresponding 3D reconstructions (red and green dots) are overlayed with the ground-truth data obtained from a Kinect sensor (gray). The color encodes the polarity, which is obtained from the event-based sensor.

To assess the overall performance of the stereo network, we recorded a dynamic scene with two neuromorphic vision sensors and sent the address-events generated by the sensors as inputs to the subsequent populations of neurons. Figure 2 shows how the network successfully solves the stereo correspondence problem, even in the case of a complex scene such as two people walking past each other at different depths. The output spikes of the stereo network were binned into 30 ms frames with *x* and *y* representing the pixel coordinates, and *d*, the pixel value. The data was then quantitatively evaluated with ground-truth recordings (see Supplementary Materials). The quantitative results are also shown in Fig. 2. The demonstration that the stereo network performs very well is evidenced by the small local average disparity error *ε*_*d*_ < 1 pixels throughout the entire duration of the scene. The disparity error remains largely constant, even at the point when the two people cross each other. At this point, the scene is dominated by large disparity gradients, which is a typical scenario where classical frame-based cooperative networks fail. Conversely, the network presented here can resolve these disambiguities by integrating not only spatial but also temporal information. This highlights how the network naturally exploits also motion cues (persons moving into different directions) to resolve the stereo correspondence problem. The consistently low disparity error also suggests that the network is very robust. Over the entire scene, a total amount of 765, 575 3D events were recorded. Using the performance metric proposed for this evaluation method, our network reached a PCM (percentage of correct matches) of 96%. A thorough analysis of different scenes is provided in the Supplementary Material.

**Figure 3:**
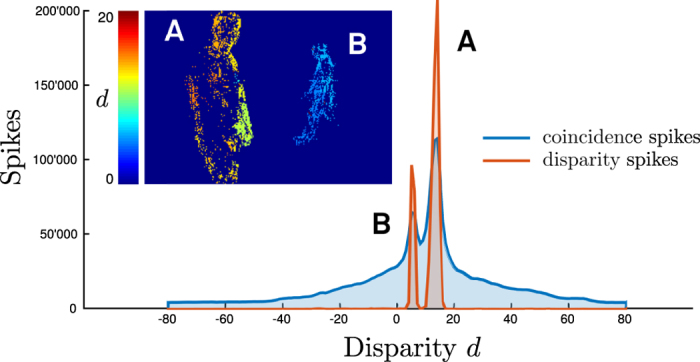
Inhibition of ambiguity in the stereo network. Spiking activity of coincidence (blue) and disparity (brown) detectors at varying disparities accumulated over the full duration of the walking scene. The inset shows a disparity map generated from a short section of the scene. The two people are labeled (**A**) and (**B**) accordingly.

Figure 3 shows a comparison of the activity of coincidence and disparity neurons for a scene in which two subjects, A and B, move at constant depths. The data shows how the responses from coincidence detectors are spread over the entire range of disparities, as would be expected when spikes are falsely matched. Conversely, the disparity detectors data shows only activity in two narrow regions, corresponding to the depths of the two subjects A and B, whereas all other disparities (which correspond to false targets) are suppressed.

### Response to dynamic random dot stereograms

**Figure 4:**
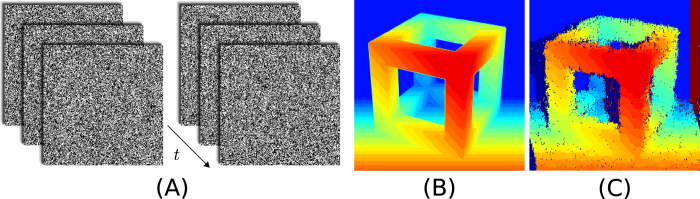
The stereo network’s response to a dynamic random dot stereogram (dRDS). (**A**) Schematic of the dRDS stimulus for the left and right eye. (**B**) Ground-truth disparity image. Disparity is encoded by color ranging from near (red) to far (blue). (**C**) Disparity map generated from accumulated responses of the network while the dRDS stimulus was presented for 1 second.

Dynamic random dot stereograms (dRDS) are commonly used in psycho-physical experiments to study the response of disparity-tuned cells in the visual system or to measure stereo acuity. We used dRDS to test the response properties of our stereo network: we generated a dRDS from a sequence of RDS, updated at 100 Hz. The initial RDS image was computed based on a disparity image of a wireframe cube (Fig. 4B) and a random noise pattern, both of which had equal dimensions of 250 × 250 pixels. Regions containing the same disparities in the disparity image were shifted in the random noise pattern accordingly. In the case of images containing varying disparities, this procedure inevitably leads to areas with undefined disparities, which are observable in the form of shadows in Fig. 4C. Subsequent RDS images were generated from the previous one in such a way that there was a 20% chance that each pixel would change polarity. Examples of three subsequent RDS are shown in Fig. 4A. The complexity of the matching problem depends on the frequency at which the RDS images are updated and the probability of a pixel changing polarity. If only a few pixels change their polarity in each consecutive image of a sequence, this could result in a trivial matching problem. This is true even if the update rate is high, assuming that coincidence detectors are tuned for very short temporal delays (such that they only respond to coincidence within a single RDS and not in-between consecutive RDS images). Here, the matching problem was guaranteed to be complex given the parameters and could not be solved trivially from the temporal information provided by the stimulus: as correspondences are only possible on lines of equal *y* coordinates (epipolar lines), the average number of potential matches between two subsequent RDS images is 0.2 · 250/2 = 25 (the division by two relates to the fact that there are individual coincidence detectors for each polarity). The final response of the network is illustrated in the form of an accumulated disparity map in Fig. 4C. Based on a qualitative comparison between the disparity map and the ground-truth data, the proposed network solves the stereo correspondence problem, even for a relatively complex dRDS containing highly varying disparity gradients.

### Neuromorphic hardware implementation

Neuromorphic hardware systems typically comprise hybrid analog/digital electronic circuits operated in the sub-threshold domain that implement faithful models of neurons and synapses^4^. Due to their low power sub-threshold operating region, their sparse activity and signal representation, and asynchronous “data-driven” nature of signal transmission they are extremely low power neural processing systems (e.g., using a few pico-Joules per spike^4^,^10^,^11^,^20^).

To validate the model proposed with a neuromorphic hardware platform we used the Reconfigurable On-Line Learning Spiking (ROLLS) neuromorphic processor device described in ref. 10. This device comprises an array of 256 analog Integrate-and-Fire neuron circuits, and 512 × 256 dynamic synapses. When an address-event is delivered to one of these synapses, the incoming voltage pulse is converted into an exponentially decaying current which is conveyed to the destination neuron and eventually summed to other currents produced by other synapses. The height of this current waveform can be set by changing the synaptic weight parameter, and the time-constant of the decaying exponential can be set by a corresponding time-constant parameter. Programmable digital circuits embedded in the arrays control the network connectivity properties, for example to implement recurrent or multi-layer networks. In particular, we used the ROLLS neuromorphic processor to implement the critical part of the model that carries out coincidence evidence integration and disparity detection by using silicon neurons which receive input address events from the coincidence detector units. To implement in neuromorphic hardware the part of the model that is composed of coincidence detection units it would be necessary to use devices with larger numbers of neurons, which are now becoming accessible^11^,^21^,^22^. As we did not have any such devices at our disposition at the time of our experiments, we implemented the coincidence detector neurons using a Field Programmable Gate Array (FPGA) device.

**Figure 5:**
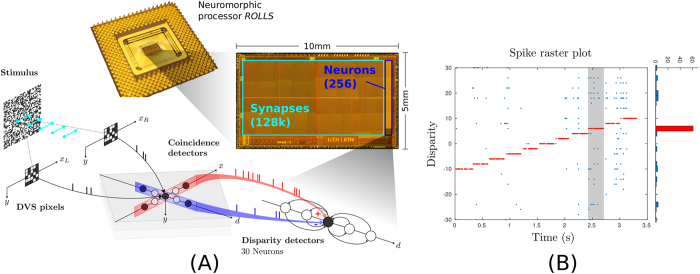
Emulation of the stereo network on a neuromorphic processor. (**A**) A RDS stimulus (printed on a chart) was moved at specified depths in front of a pair of dynamic vision sensors. The depth of the stimulus was detected by 30 disparity neurons covering an equally spaced disparity range from −10 to +10. Disparity detectors were emulated by a neuromorphic processor. (**B**) Spike event output of the disparity neurons during stimulus presentation. The spikes from the neuron encoding the true disparity are colored red, while all the others are colored blue. The histogram shown on the right represents the distribution of disparity spikes for the time where the stimulus was at a fixed disparity *d* = 6 (indicated by the grey shaded region).

The experimental setup is illustrated in Fig. 5A. An RDS stimulus was printed on a chart and moved across multiple depths in a fronto-parallel plane to the rectified stereo setup comprising the two Dynamic Vision Sensors (DVS)^23^. The pixels of the two vision sensors were grouped to form 30 binocular 21 × 21 receptive fields and spread along their central epipolar lines. These inputs were projected in parallel to the FPGA coincidence detector units. The output of these units were then sent to the silicon neurons on the neuromorphic processor in a way to encode 30 disparities equally distributed along a line of constant cyclopean position. Mutual inhibition among disparity neurons was implemented using on-chip recurrent inhibitory connections.

The response of this stereo vision systems is shown in the form of a spike raster plot in Fig. 5B. The stimulus was presented at equally spaced disparities ranging from −10 to +10. The spikes from the neuron encoding the true disparity are colored red, while all the others are colored blue. The network successfully detects the correct disparity of the stimulus in the entire range, as indicated by the red spike trains. While a certain disparity detector is active, the others are strongly suppressed by the recurrent inhibitory connections.

For comparison, a classical stereo vision system involving local stereo matching based on the sum-of-absolute-differences (SAD) could be implemented on a conventional micro-controller. In this case, the computational cost would be heavily dominated by the calculation of the SAD. An operational primitive of the SAD algorithm is the calculation of the absolute difference of two intensities followed by a sum operation. The number of such operations grows with the input data rate (i.e., the camera frame-rates), irrespective of the scene contents. In the stereo network we propose, the equivalent operational primitive is a coincidence detection followed by a spike integration operation (only when there are coincidences). The number of these operations depends on the response of the DVS to the contents of the scene. These considerations allowed us to estimate the difference between numbers of operations made with the two different implementations (see the Methods section for details), and to derive an estimate of the power budget based on energy measurements of these primitive operations. In Table 1, we present these comparison estimates, showing how a micro-controller implementation of a SAD stereo vision algorithm run at a temporal resolution that is comparable to the one used by our method (i.e., at 151 Hz) would consume approximately an order of magnitude more power than an spiking stereo neural network (SSNN) implementation done using the ROLLS neuromorphic technology. As the ROLLS chip was fabricated using an old technology, the SSNN energy cost per operation is significantly higher than that used by a state-of-the-art micro-controller. In this case, the SSNN advantage in total power consumption is solely due to the data-driven event-based processing nature of the visual signal. Analogous neuromorphic processors fabricated in a state-of-the-art 28 nm VLSI process technology would have even smaller energy cost per primitive operation^11^.

This comparison was made using the moving random dot charts, similar to those shown in Fig. 4A, with very high spatial contrast. In real-world applications the data rate produced by the DVS would be significantly lower, leading to even lower power consumption figures.

## Discussion

In this paper we proposed a spiking neural network model that solves the stereo correspondence problem efficiently by exploiting an event-based representation, and that is amenable to neuromorphic hardware implementations. The network expects in input visual data in the form of asynchronous events produced by neuromorphic vision sensors and processes these address-events in a data-driven manner using computational operators that can be implemented with synapse and neuron models. Although heavily inspired by neuroscience of the early visual pathway and work on stereopsis, this network it nonetheless an abstract simplification of biological stereo-vision systems. One fundamental difference is that the network proposed operates using exclusively precisely-timed temporal contrast events, as measured directly from the neuromorphic vision sensors, which model only the *transient* responses of retinal cells (i.e., of the Y-ganglion cells), without including the sustained ones. As these transient responses produce single events, their precise timing plays a crucial role in the stereo correspondence process. In contrast, the vast majority of computational neuroscience models of stereopsis are based on mean firing rates, and do not rely on the precise timing of spikes. In these models the behavior of V1 neurons are described by tuning functions that predict the neuron’s firing rate in response to a given stimulus in its receptive field. Such tuning functions were found to be well predicted by Gabor filters, which can explain characteristics of V1 cells such as orientation and spatial frequency tuning. Accordingly, stereopsis models are based on binocular energy neurons that combine monocular Gabor filters and predict the responses of disparity-tuned binocular cells in V1^24^. The mechanism of phase and position disparity are direct consequences of the way in which receptive fields and tuning functions are described. While these models explain many aspects of the physiology of stereopsis, they do not make explicit use of important features of neural systems such as their *temporal dynamics* or the precise spike-timing of the neurons. By contrast, the model we presented does not incorporate orientation, frequency tuning, or phase and position disparity mechanisms. These characteristics are based on the perception of *spatial contrast*, whereas the proposed model solely responds to *temporal contrast*. Different interpretations of the model we presented are possible, based on architectural and functional considerations: For example, this spike-timing model could be functionally combined with the rate-based energy model: higher order disparity detectors could integrate transient responses of energy neurons rather than events from neuromorphic vision sensors. In this case, an address-event would not represent a spike of a retinal ganglion cell, but the output of a cortical simple cell, and the model could combine the best features of both approaches. Alternatively, our network could indeed represent an independent module operating exclusively on the fast transient pathway, to implement a fast and coarse stereopsis system which would coexist in parallel with a more precise and substantial stereopsis process, as described by the disparity energy models. Such a coarse stereo process could be involved for example in vergence eye movement as part of the magnocellular system.

Correlation models of stereo correspondence are prevalent in neuroscience^25^,^26^. Disparity detectors at early stages are believed to be tuned to small patches of uniform fronto-parallel disparities. When combined, they could be used to perceive more complex disparity structures in higher areas^27^. This proposition conforms with the first observation made by Marr and Poggio^19^. After describing the prevalence of smoothly distributed disparities in natural scenes, the authors propose a rule of excitation among cells of equal disparities in their cooperative network. In the method described here, this mechanism was adapted and supplemented with an inhibiting mechanism, suggesting that the proposed neurons perform an approximation of local covariance of spatiotemporal visual information (see Supplementary Material for details). Spatiotemporal information strengthens the correlation even in the case of non-fronto-parallel disparities (when the motion is fronto-parallel). In other words, the stereo network naturally exploits motion cues to overcome the limits of stereo matching based on spatial correlation alone. This is achieved simply by using a different concept to encode visual information. In neuroscience, it is well known that motion cues can play a crucial role in solving the correspondence problem. However, it is not clear how and where they are integrated in the brain. Experimental studies in macaque monkeys and humans suggest that the primate brain needs to integrate several cues ranging from low to high cortical levels in order to solve the correspondence problem^28^,^29^. Among these cues, motion (that is integrated in mid-level visual areas) is one of the most important.

Recent studies support the idea that V1 neurons play a more important role in discriminating correct and false disparities and that the widely accepted disparity energy model needs to be revised. A computational study proposed that by adding suppressive elements to the energy model, responses to false matches may be attenuated^30^. This has been confirmed with experimental results from the V1 neurons of monkeys^31^. Interestingly, the work in hand draws the same conclusion from an entirely computational approach, without any prior knowledge of the physiological findings. The additional use of inhibiting elements in the proposed disparity neuron model means that in effect, the neurons compute a form of correlation that behaves similarly to the covariance of interocular spatiotemporal information. Thus, the neurons clearly show attenuated responses to false targets. In the model presented here, the elements of inhibition are coincidence neurons from the plane of constant cyclopean position *E*_*x*_. These coincidence detectors receive input from retinal cells that are positioned relatively similarly in the two retinas to those that provide the input to two biological cells: phase-detectors in anti-phase and position-detectors with different preferred disparity. In stereopsis research, exactly these type of detectors have been proposed to represent the suppressive mechanism in V1 neurons that helps to solve the correspondence problem^30^,^31^,^32^.

A few psychophysical illusions exist that are related to the process of stereopsis such as the Pulfrich effect and the double-nail illusion. The double-nail illusion occurs when two identical objects (for example two nails) are viewed straight ahead at reading distance at the same position, but are separated in depth by a few centimeters. The two objects are perceived as if aligned side-by-side (false targets) instead of one being behind the other (true targets). This illusion can be explained by any model based on correlation measures, such as the one we propose or the original Marr and Poggio model. However, unlike other models, our model is also compatible with human perception when the two objects are moving: if the two objects move sideways at constant depth, they are typically perceived at the correct positions (true targets), even at disparity gradients that exceed the limit of human stereopsis. As the model we propose naturally exploits motion cues, it would correctly detect the true targets as well. As a logical consequence, if the two objects were separated in terms of horizontal position but aligned at the same reading distance and moved alternately back and forth, our model would predict that the false targets, which would be moving sideways at different depths, would be perceived. This would be an interesting psychophysical illusion that could be easily tested on humans.

One of the most important features of the nervous system is its ability to implement plasticity. This enables biological neural processing system with adaptive and learning abilities that are used to change and tune its parameters. The stereo network that we implemented in this work does not have these abilities. Indeed, the network topology, and its weights have been determined with a synthesis procedure and a calibration method that allowed us to assume that the coincidence detectors received inputs from pairs of retinal cells which would correspond to a true physical location in space (i.e., assuming stereo rectification of the inputs). Indeed, plasticity would be extremely useful in such a delicate calibration process, in order to automatically rectify the inputs. In previous work^33^ we have shown how these plasticity mechanisms could exploit the interocular temporal coincidence to learn in an unsupervised way the epipolar geometry of the scene (rectification). Such unsupervised learning could be directly incorporated into our model, at the level of the synaptic connections that link the retinal cells and the coincidence detector units.

Although tightly linked to neuroscience, we expect the most significant impact of our model to be in the field of machine vision. Today’s machine vision processing systems face severe limitations imposed both by the conventional sensor front-ends (which produce very large amounts of data, as sequences of frames, but with fixed sampled frame-rates), and the classical von Neumann computing architectures (which are affected by the memory bottleneck^18^,^34^ and require high power and high bandwidths to process continuous streams of images). The emerging field of neuromorphic engineering has produced efficient event-based sensors, that produce low-bandwidth data in continuous time, and powerful parallel computing architectures, that have co-localized memory and computation and can carry out low-latency event-based processing. This technology promises to solve many of the problems associated with conventional technologies in the field of machine vision. However, so far the progress has been chiefly technological, whereas related development of event-based models and signal processing algorithms has been comparatively lacking (with a few notable exceptions). This work elaborates an innovative model that can fully exploit the features of event-based visual sensors. In addtion, the model can be directly mapped onto existing neuromorphic processing architectures. The results show that the full potential is leveraged when single neurons from the stereo network are individually emulated in parallel. In order to emulate the full-scale stereo network, however, efficient neuromorphic hardware device capable of emulating large-scale neural networks are required. Although a few promising approaches already exist^11^,^21^,^22^, large-scale, but compact, re-configurable, and low-power systems remain a challenge in neuromorphic engineering.

## Methods

### Neuromorphic silicon retina

**Figure 6:**
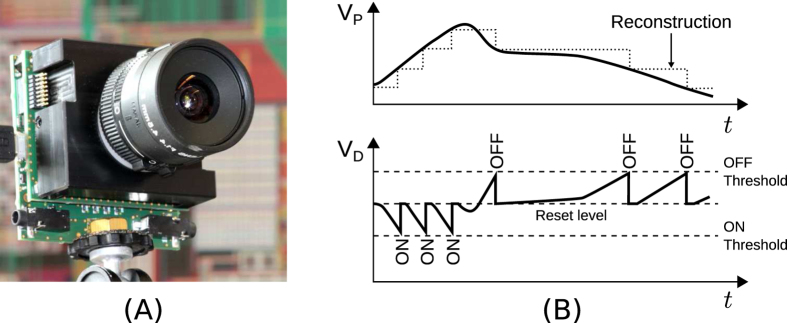
The silicon retina. (**A**) DAVIS sensor. (**B**) Principle of ON and OFF spikes generation of DVS pixels, adapted from ref. 23. Top: the evolution of pixel’s voltage *V*_*p*_ proportional to the log intensity. Bottom: the corresponding generation of ON (voltage increases above change threshold) and OFF (voltage decreases) events, from which the evolution of *V*_*p*_ can be reconstructed.

Unlike classical frame-based cameras, biological retinas encode visual information more efficiently, in a less redundant manner. The underlying principle is an asynchronous sampling strategy, implying that biological retinas acquire not only spatial contrast at discrete points in time but continuously sense spatial and temporal changes. The pixels of so called neuromorphic, event-based vision sensors only send out information when they are exposed to a change in illumination generating a compressed informative output. Compared to classical cameras, this readout reduces redundancy allowing a fast acquisition with low latency and high temporal resolution. The experiments shown in this work were conducted with the DAVIS^35^ which is an extension of the DVS featuring a higher spatial resolution of 240 × 180 pixels and an additional APS readout (not used in this work). The DVS output consists of events each of which reflecting a change in the log intensity at the given spatial position. The polarity of those events represents whether the intensity increased (ON event) or decreased (OFF event). As shown by Fig. 6B, an event is immediately generated when the change in log intensity exceeds a given threshold (typically 15% of contrast) being completely independent of a synchronous readout clock as it is used in conventional cameras. The timing of events can therefore be conveyed with a very accurate temporal resolution of approximately 10 *μs*, allowing an “effective frame rate” typically in the range of several kilohertz. A further advantage of this sensor is that pixels are not bound to a global exposure time, allowing them to independently adapt to local scene illumination resulting in high dynamic range from 0.1 lux to over 100 klux. In order to process data on a computer, a dedicated FPGA acquires the events from the sensor and attaches a digital timestamp. The synchronized data is then transmitted over a USB connection to the host computer for processing. Alternatively, events can also be send out in real-time via an asynchronous, digital bus in order to directly connect it to a neuromorphic processor for example.

### Spiking stereo neural network model

Each neuron in the network is uniquely assigned a horizontal and vertical cyclopean coordinate *x* and *y*, as well as a disparity coordinate *d*. With the definition of 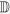 being the one-dimensional disparity space, then the three coordinates represent a point in the 3D disparity space 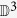, which corresponds to the neuron’s cognitive representation of a location in 3D space. We can thus define the map 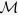 which transforms retinal image coordinates to disparity space as


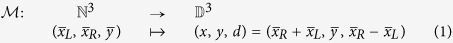


where 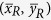 and 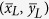 are rectified pixel coordinates of the retinal input neurons. In order to implement a neural coincidence detection mechanism, the proposed network uses neurons with leaky-integrate-and-fire (LIF) dynamics^36^. The membrane potential *v*_*c*_(*t*) of a LIF coincidence neuron is described by the following equation


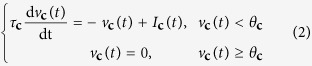


where the time constant *τ*_**c**_ determines the neuron’s leak and *θ*_**c**_ the threshold at which the neuron fires. A coincidence neuron receives input from a pair of epipolar retinal cells, which can be described as a sum of spikes


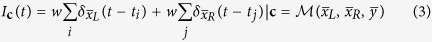


where the indices *i* and *j* indicate the spike times of the retinal cells 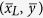 and 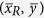 respectively. The proposed disparity detectors aggregate evidence from the responses of simple coincidence detectors. The disparity detectors are also modeled using LIF neuron dynamics, but with a distinct time constant *τ*_**d**_ and a firing threshold *θ*_**d**_:


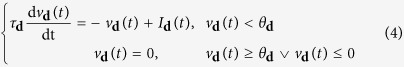


The input of the disparity detector at **d** = (*x*_*d*_, *y*_*d*_, *d*_*d*_) combines the outputs from coincidence detectors within bounded planar excitatory and inhibitory regions in disparity space 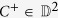 and 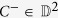 respectively and outputs from disparity detectors in the mutually inhibitory region 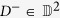:


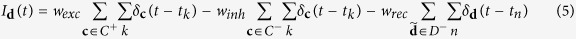


where *k* represents the index of the spike times of coincidence neuron **c**, while *w*_*exc*_ and *w*_*inh*_ are constant excitatory and inhibitory weights. The regions *C*^+^ and *C*^−^ are squared windows in the plane of constant disparity *E*_*d*_ and the plane of constant horizontal cyclopean position *E*_*x*_, which are defined relative to the disparity detector’s spatial representation **d**. The index *n* represents the spike times of disparity neuron 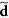 and *w*_*rec*_ is a constant inhibitory weight (mutual inhibition of disparity detectors). The region *D*^−^ is defined by the two lines of sight (one from each retina):


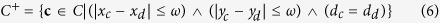



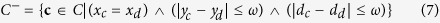



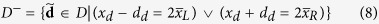


The final output of the network is then obtained from a combination of coincidence and disparity responses such that a disparity spike is only considered valid when immediately preceded by a coincidence spike representing the same location in disparity space (see Supplementary Material for more details).

### Network simulation

For our simulations we used the output of 180 × 180 pixels from each sensor. This led to a population of 2 × 180^3^ coincidence detectors (polarities where treated with separate detectors). With another 180^3^ disparity detectors, the total network initially incorporated more than 17 million neurons. However, for a natural scene within 5 m distance from the camera, the disparity is limited to the range (0, 40) so the total number of required neurons was reduced to less than 4 million. The simulation code was entirely written in C and in a completely event-based fashion, meaning that the membrane potential of a specific neuron was only updated when it received an input leading to a very efficient implementation. For each neuron the current membrane potential and the time of the last update needed to be stored in memory ending up in an occupancy of about 30 megabytes. In the 4 seconds walking scene, roughly 1.2 million input events were processed with a total simulation run time of about 30 seconds on a single core of an i7 CPU running at 3.40 GHz.

### Comparing stereo correspondence performance of traditional and neuromorphic hardware

Conventional SAD algorithms for stereo matching typically involves many processing steps. However the step that dominates the algorithm complexity is the calculation of the sum of absolute differences. Typically, a three-dimensional map of disparity space is calculated, in which each entry contains the absolute difference of intensity of the corresponding image pixels at a given disparity. The sum can then be easily computed across constant disparities. This three-dimensional map is then updated for each pair of images, with every new frame. As a consequence, the algorithm complexity is proportional to *n*^2^*Df*_*s*_, where *n*^2^ is the dimension of the disparity image, *D* the depth resolution and *f*_*s*_ the sampling rate. Typically, with this method, a subtraction, rectification and addition needs to be done at each step. For the sake of simplicity, we don’t consider in the comparison, the extra steps required to identify the highest SAD entry in the map. In our stereo-correspondence model the disparity map is represented by the population of coincidence neurons, which are updated only when the afferent pixels produce address-events. This update rate determines the network complexity and is proportional to *n*^2^*d*^2^*Dr*, where *d* can represents the edge density of the visual input and *r* is the rate by which the edges change. This is a highly simplified expression and assumes that intensity changes are caused by moving edges. It becomes immediately evident that for a rate of change *r* that matches the sampling rate *f*_*s*_, our stereo network requires a factor *d*^2^ less operations. In the context of the stereo network, the generation of a coincidence spike and its propagation to the disparity detectors is considered an operation. This is approximately equivalent to the SAD operation (subtraction, rectification, addition) as concluded from our network simulations and detailed analysis.

For the estimation of the power consumption of the SAD algorithm we considered a state-of-the low power art micro-controller, such as the STM32F7 series, which has power consumption figures rated as low as 0.33 mW/DMIPS. In this calculation we assumed that a SAD operation is implemented with 3 DMIPS (which is the bare minimum not considering any parallel processing). We scaled the number of operations to match the described scenario, considering a depth resolution of 30 levels, computed within a field of view of 21 × 21 pixels.

In the spiking stereo neural network (SSNN) the number of operations corresponds to the amount of input coincidence spikes. This number was calculated from the input spikes produced by the DVS and estimated edge density of the visual scene. Using this estimate we could calculate the rate of change of edges (*r* = 151 Hz), which defines the temporal resolution at which the two approaches should be compared.

For the estimation of the power consumption on neuromorphic hardware implementation, we considered the energy used for both generation and the routing of address-events. These figures were derived from measurements taken from a more recent design of a neuromorphic processor comprising the same circuits of the ROLLS chip, and using the same 180 nm CMOS technology: the energy cost of a single spike generation is 883 pJ and the routing of a spike is 360 pJ resulting in a total of 1.243 nJ per primitive operation. Using more advanced technology nodes, such as the 28 nm one used for recently proposed neuromorphic processors^11^, these figures would be even smaller. We are currently developing a neuromorphic processor chip that can support the network proposed in this paper, using a 28 nm process, and have estimated energy figures of 50 nJ for the spike generation, and 147 nJ for routing the event to its destination^20^, resulting in a dramatically smaller total cost of 197 pJ per primitive operation. The results of Table 1 summarize this analysis.

In both SAD and SSNN calculations, we considered only the cost of the core operations, neglecting the costs of peripheral functionality such as I/O operations.

## Additional Information

**How to cite this article:** Osswald, M. *et al*. A spiking neural network model of 3D perception for event-based neuromorphic stereo vision systems. *Sci. Rep.* **7**, 40703; doi: 10.1038/srep40703 (2017).

**Publisher's note:** Springer Nature remains neutral with regard to jurisdictional claims in published maps and institutional affiliations.

## Supplementary Material

Supplementary Material

## Figures and Tables

**Table 1:** Estimated power consumption of classical hardware implementation versus neuromorphic hardware implementation.

	Update rate	Operations/s	Energy per operation	Est. power consumption
SAD	30 Hz	397 K	0.99 nJ	393 *μ*W
SAD	151 Hz	2.00 M	0.99 nJ	1.99 mW
SSNN (180 nm)	151 Hz	147 K	1.243 nJ	182 *μ*W
SSNN (28 nm)	151 Hz	147 K	0.197 nJ	29 *μ*W
