# Corrigendum: A spiking neural network model of 3D perception for event-based neuromorphic stereo vision systems

**DOI:** 10.1038/srep44722

**Published:** 2017-03-16

**Authors:** Marc Osswald, Sio-Hoi Ieng, Ryad Benosman, Giacomo Indiveri

Scientific Reports 7: Article number: 4070310.1038/srep40703; published online: 11 12 2017; updated: 03 16 2017

The Authors neglected to cite a previous study related to biological stereo vision systems. The additional reference is listed below as reference 1, and should appear in the text as below.

In the Introduction section,

“Here we present a novel approach to the stereo correspondence problem, inspired by biological stereo vision systems, which is compatible with ultra low latency and low power neuromorphic hardware technologies^4^”.

should read:

“Here we present a novel approach to the stereo correspondence problem, inspired by biological stereo vision systems and the pioneering work of Mahowald^1^. Our approach is compatible with ultra low latency and low power neuromorphic hardware technologies^4^”.
