# Supplementary material for: A spiking neural network model of 3D perception for event-based neuromorphic stereo vision systems
Source: Sci Rep. 2017 Jan 12;7:40703. doi: 10.1038/srep40703 (PMC5227683; doi:10.1038/srep40703)
Supplement: Supplementary Material [file srep40703-s1.pdf]

Supplementary Material

A spiking neural network model of 3D perception for event-based neuromorphic stereo vision systems

Marc Osswald[1], Sio-Hoi Ieng[2], Ryad Benosman[2], and Giacomo Indiveri[1,*]

[1]Institute of Neuroinformatics, University of Zurich and ETH Zurich, Zurich, Switzerland
[2]Université Pierre et Marie Curie, Institut de la Vision, Paris, France
[*]giacomo@ini.uzh.ch

S1 The spiking stereo neural network

This section provides a detailed explanation of the spiking stereo neural network.

S1.1 The coordinate system of the network

Similar to place cells in rats, each individual neuron in the network acts as a cognitive representation of a unique location in 3D space. The way in which this 3D space should be sampled by the neurons is not a trivial consideration. Consider a population of neurons for example, which represent uniformly distributed points in absolute-world coordinates. In this case, the way in which the retinas — here referred to as sensors — project into the network involves dynamic mapping, which is determined by the projective geometry of the sensor arrangement. Such mapping not only requires precise calibration, it also depends on the dynamic parameters of the system such as the focal length and convergence angle between the sensors. An alternative mapping technique could involve assigning a fixed pair of points, one from the image plane of each sensor, to each neuron. This neuron would then represent the spatial location of the point of intersection of the two lines of sight from its associated image points. Accordingly, the neuron would encode depth relative to the common fixation point of the sensors. The advantage of relating the neurons to the input rather than fixing their representation in space is that it allows for static mapping between the input and network, even if the sensor arrangement is varied. The exact process of this mapping mechanism and its implications for how the network samples the 3D space is explained as follows.

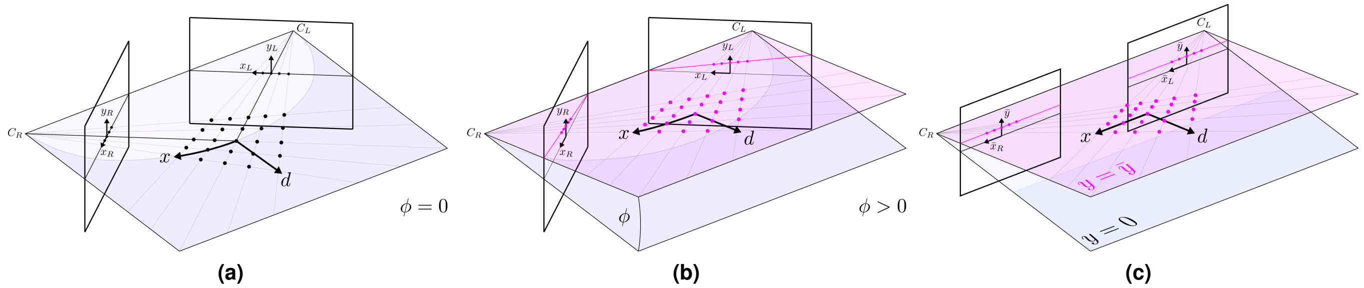

Figure S1. The coordinate system of the network and representation of disparity space. (a) The general sensor arrangement, showing the epipolar plane that is perpendicular to the image planes. (b) The same sensor arrangement, showing an inclined epipolar plane. (c) The same sensor arrangement with rectified image planes.

In Fig. S1a, a general stereo setup is shown comprising two sensors located at C_R and C_L and their image planes, each with its own image coordinates (x_R, y_R) and (x_L, y_L) respectively. The plane, spanned by any 3D point and the centers of the cameras is known as the *epipolar plane*. All points lying on an epipolar plane project to exactly one line in each image, the *epipolar line*. The epipolar lines are formed by the intersection of the epipolar plane with the image planes. Here, different epipolar planes are represented according to their inclination ϕ. The shaded blue plane indicates the horizontal epipolar plane as $\phi = 0$. In this example, five image points lying on the horizontal epipolar lines and their 25 correspondences in 3D space, forming a distorted

array, are shown. Within this array, indices x and d along the diagonals are introduced. The indices are directly derived from the image coordinates:

$$\begin{aligned} x &= x_R + x_L \\ d &= x_R - x_L \end{aligned} \quad (1)$$

where x is referred to as the *horizontal cyclopean coordinate* and d as the *disparity coordinate*. Each epipolar plane now forms a layer of the network. Then, each neuron of the network can be uniquely described by the triplet (x, ϕ, d). In order to get an impression of how the representations of the neurons are distributed in 3D space, a different layer of the network, corresponding to an inclined epipolar plane ($\phi > 0$) is shown in Fig. S1b. The challenge that remains is to define the relationship between the image coordinates and the inclination ϕ of the epipolar plane, as this depends on the poses of the sensors. This problem is resolved using the concept of image rectification, which is graphically illustrated in Fig. S1c. Image rectification is a homographic transformation that re-projects the image planes so that they are coplanar. The image points are then expressed in *rectified coordinates*, $(\bar{x}_R, \bar{y}_R)$ and $(\bar{x}_L, \bar{y}_L)$. Rectification requires a number of correspondences among the points and is a standard procedure in machine vision. Rectified coordinates entail corresponding epipolar lines which have the same constant vertical coordinate $\bar{y}_L = \bar{y}_R = \bar{y}$. The inclination of the epipolar plane is directly related to $\bar{y}$ and provides a better representation of disparity space. Thus, a new representation is obtained by using rectified image coordinates and redefining $d = \bar{x}_R - \bar{x}_L$, $x = \bar{x}_R + \bar{x}_L$ and $y = \bar{y}$ accordingly. A unique map $\mathscr{M}$ can then be derived, which is invariant to sensor pose, and which assigns a neuron to each epipolar pair of rectified image coordinates. This neuron can be described by the triplet (x, y, d):

$$\begin{aligned} \mathscr{M}: \quad \mathbb{N}^3 &\rightarrow \mathbb{D}^3 \\ (\bar{x}_L, \bar{x}_R, \bar{y}) &\mapsto (x, y, d) = (\bar{x}_R + \bar{x}_L, \bar{y}, \bar{x}_R - \bar{x}_L) \end{aligned} \quad (2)$$

where (x, y, d) are the network coordinates and their range is the *disparity space* $\mathbb{D}^3$. Finally, each neuron is uniquely assigned a horizontal and vertical cyclopean coordinate x and y, as well as a disparity coordinate d. Together, these coordinates represent a point in disparity space $\mathbb{D}^3$, which corresponds to the neuron's cognitive representation of a location in 3D space. The absolute-world coordinates of this location are determined by the intersection of the lines of sight from the pair of rectified image points, which can be derived from the network coordinates by means of the inverse mapping function $\mathscr{M}^{-1}$.

S1.2 The architecture of the network

A spiking neural network is proposed that extends the classical cooperative network by adopting the previously described mechanism of stereo correspondence based on temporal and spatial compliance. An abstract view of the entire network architecture is given in Fig. S2a. The retinal cells (or pixels of the sensors) are represented by the populations L and R and serve as the input to the network. Their size is indicated by n^2, as the sensors consist of two-dimensional pixel arrays. L and R excite a population of neurons C, referred to as the "coincidence detectors". The size of the population C scales cubically because the neurons within it encode coincidences that occur in disparity space. Lastly, another population of neurons D, termed the "disparity detectors", pool responses from C in mixed excitatory and inhibitory manner. In order to signal only correct disparities, the recurrent inhibitory connections among neurons in D implement a winner-takes-all mechanism designed to suppress disparity responses to false targets. A detailed view of a horizontal layer of the network is illustrated in Fig. S2b. Following on from the rationale for using temporal images, spiking retinal cells are used as inputs. Each spike represents a change in illumination at a specific spatial position at a particular time. For each pair of corresponding horizontal lines of retinal cells from L and R, a horizontal layer of neurons in C signals temporally coinciding spikes. The cells in C are arranged according to the previously defined disparity coordinate system of the network. Thus, each cell has a unique spatial representation in disparity space (x, y, d) (only x and d are shown) such that each spike provides evidence for a potential target at the corresponding real-world disparity position. Thus, the complete population of coincidence detectors encodes all potential targets (true and false disparities). The disparity detectors implement a binocular correlation mechanism, which is realized by integrating the responses from coincidence detectors within the planes of constant disparity E_d and cyclopean position E_x. Activity in E_d constitutes supporting evidence for true matches (excitation of disparity detector), whereas activity in E_x denotes countervailing evidence (inhibition of disparity detector). Finally, the uniqueness constraint is enforced by mutual inhibition of disparity detectors that represent spatial locations in the same line of sight.

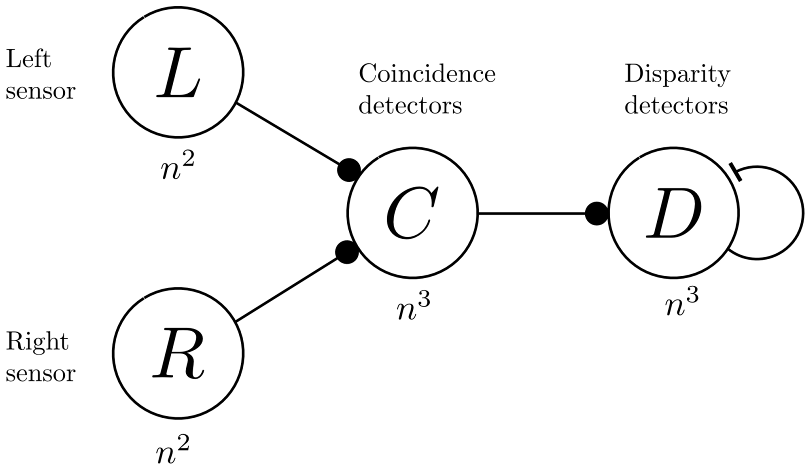

(a)

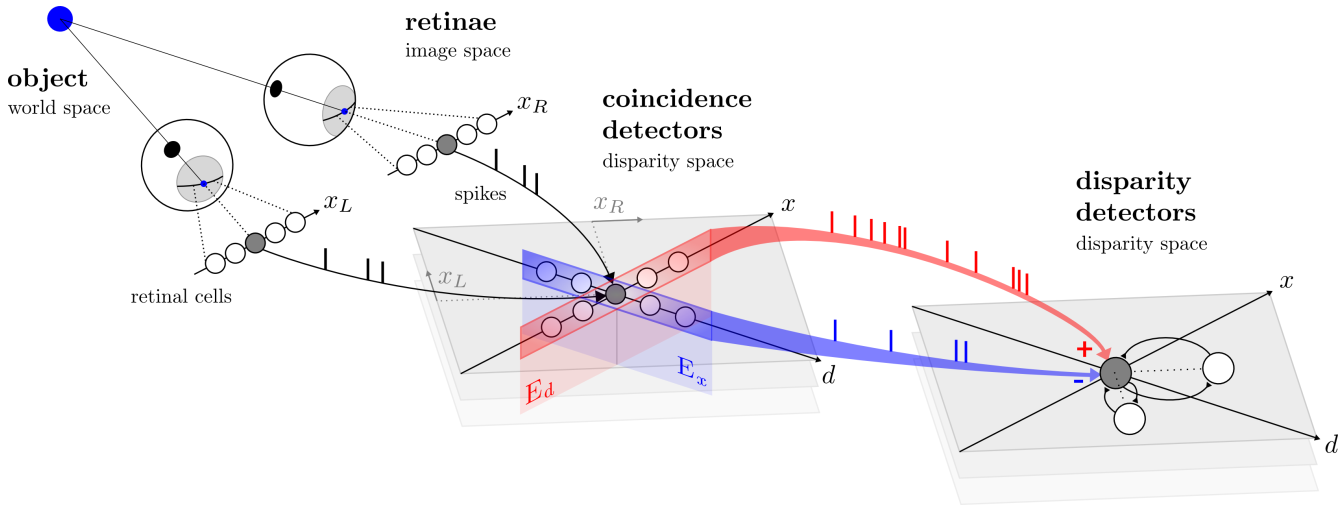

(b)

Figure S2. The spiking stereo network. (a) Abstract view of the network's architecture. (b) Detailed view of a horizontal layer of the network. An object is sensed by two eyes and accordingly projected onto their retinal cells. The spiking output of these cells is spatiotemporally correlated (coincidence detectors) and integrated (disparity detectors). The final output encodes a representation of the original scene in disparity space. For the sake of visibility, only a horizontal line of retinal cells, at fixed vertical cyclopean position y, is considered. The corresponding coincidence and disparity detector units, hence, lie within a horizontal plane. Again, only a few units are shown here whereas in the complete network, the units would be uniformly distributed over the entire plane. The shaded planes indicate how the network expands vertically over y.

S1.3 Simple coincidence detectors

In order to implement a neural coincidence detection mechanism, the proposed network uses neurons with leaky-integrate-and-fire (LIF) dynamics.[1] The membrane potential $v_\mathbf{c}(t)$ of a LIF coincidence neuron is described by the following equation

$$\begin{cases} \tau_\mathbf{c} \dfrac{\mathrm{d}v_\mathbf{c}(t)}{\mathrm{d}t} = -v_\mathbf{c}(t) + I_\mathbf{c}(t)\,, & v_\mathbf{c}(t) < \theta_\mathbf{c} \\ v_\mathbf{c}(t) = 0\,, & v_\mathbf{c}(t) \geq \theta_\mathbf{c} \end{cases} \tag{3}$$

where the time constant $\tau_\mathbf{c}$ determines the neuron's leak and $\theta_\mathbf{c}$ the threshold at which the neuron fires. A coincidence

neuron receives input from a pair of epipolar retinal cells, which can be described as a sum of spikes

$$I_{\mathbf{c}}(t) = w\sum_i \delta_{\bar{x}_L}(t-t_i) + w\sum_j \delta_{\bar{x}_R}(t-t_j) \quad \Bigg| \quad \mathbf{c} = \mathscr{M}(\bar{x}_L, \bar{x}_R, \bar{y}) \tag{4}$$

where the indices i and j indicate the spike times of the retinal cells $(\bar{x}_L, \bar{y})$ and $(\bar{x}_R, \bar{y})$ respectively. A single spike is modeled with the Dirac function $\delta(t)$. For the obvious reason of symmetry, the synaptic weights w are equally sized for both inputs. The subscript vector $\mathbf{c} = (x_c, y_c, d_c)$ corresponds to the neuron's unique spatial representation in disparity space, explicitly defined by the previously introduced map $\mathscr{M}$. If it is assumed that spikes from the same retinal cell are temporally well separated, such that a preceding spike only has a marginal effect on the membrane potential at the time of a current spike, then the neuron's *sensitivity* $S_{\Delta T}$ to interocular temporal delays can be derived as:

$$S_{\Delta T} = \tau_{\mathbf{c}} \ln\left(\frac{1}{\frac{\theta_{\mathbf{c}}}{w} - 1}\right), \quad 1 < \frac{\theta_{\mathbf{c}}}{w} \leq 2 \tag{5}$$

The sensitivity of a coincidence detector is the range of interocular temporal delays between two spikes from an epipolar pair of retinal cells within which the neuron is responsive. The ratio $\frac{\theta_{\mathbf{c}}}{w}$ is chosen so that while two retinal spikes can trigger a response, a single spike will not. It is difficult to select an appropriate time constant $\tau_{\mathbf{c}}$ because it directly affects the sensitivity of the neuron. If the sensitivity is too high, the neuron will be selective to long interocular temporal delays, which will increase the number of coincidences associated with ambiguous disparities. On the other hand, if the sensitivity is too low, the beneficial effect of global support from distant targets with varying disparity will be reduced.

S1.4 Complex disparity detectors

Similarly to complex cells in the brain, the proposed disparity detectors aggregate evidence from the responses of simple coincidence detectors. The disparity detectors are also modeled using LIF neuron dynamics, but with a distinct time constant $\tau_{\mathbf{d}}$ and a firing threshold $\theta_{\mathbf{d}}$:

$$\begin{cases} \tau_{\mathbf{d}} \dfrac{\mathrm{d}v_{\mathbf{d}}(t)}{\mathrm{d}t} = -v_{\mathbf{d}}(t) + I_{\mathbf{d}}(t)\,, & v_{\mathbf{d}}(t) < \theta_{\mathbf{d}} \\ v_{\mathbf{d}}(t) = 0\,, & v_{\mathbf{d}}(t) \geq \theta_{\mathbf{d}} \end{cases} \tag{6}$$

The input of the disparity detector at $\mathbf{d} = (x_d, y_d, d_d)$ combines the outputs from coincidence detectors within bounded planar excitatory and inhibitory regions in disparity space $C^+ \in \mathbb{D}^2$ and $C^- \in \mathbb{D}^2$ respectively:

$$I_{\mathbf{d}}(t) = w_{exc} \sum_{\mathbf{c}\in C^+} \sum_k \delta_{\mathbf{c}}(t-t_k) - w_{inh} \sum_{\mathbf{c}\in C^-} \sum_k \delta_{\mathbf{c}}(t-t_k) \tag{7}$$

where k represents the index of the spike times of coincidence neuron $\mathbf{c}$, while w_{exc} and w_{inh} are constant excitatory and inhibitory weights. The regions C^+ and C^- are squared windows in the plane of constant disparity E_d and the plane of constant horizontal cyclopean position E_x, which are defined relative to the disparity detector's spatial representation $\mathbf{d}$:

$$C^+ = \{\mathbf{c} \in C \mid (|x_c - x_d| \leq \omega) \wedge (|y_c - y_d| \leq \omega) \wedge (d_c = d_d)\} \tag{8}$$

$$C^- = \{\mathbf{c} \in C \mid (x_c = x_d) \wedge (|y_c - y_d| \leq \omega) \wedge (|d_c - d_d| \leq \omega)\} \tag{9}$$

where ω is half of the window size. The synaptic weights should be chosen such that they are inversely proportional to the sizes of C^+ and C^-. Since C^+ and C^- are of equal size, it is suggested that $w_{exc} = w_{inh}$. The time constant of the disparity neurons determines how evidence from the past is weighted. Making an appropriate choice of $\tau_{\mathbf{d}}$ is dependent on the stimuli but in general, it is significantly larger than $\tau_{\mathbf{c}}$.

S1.5 Variants of disparity detectors

Ideally, the proposed disparity detectors would compute the spatiotemporal cross-correlation of interocular temporal images. However, cross-correlation is a complex computation that involves the calculation of the covariance followed by normalization. The responses of the proposed disparity detectors are clearly not normalized, which can be easily observed by comparing the case of a single coincidence in C^+ with the case of multiple coincidences in C^+. Assuming there are no coincidences in C^- in both cases, the latter results in a stronger response while the cross-correlation coefficient would be ideal ($\rho = 1.0$) in both scenarios. For the purposes of this project, it is argued that it is sufficient for the proposed disparity detectors to compute a covariance-like measure, while the problem of non-normalized responses is addressed by mutual inhibition (see next section). To study the computation performed by the disparity neuron, various types of detectors have been examined. Two types of complex disparity detectors are proposed, each characterized by their receptive fields:

$$\mathrm{RF}_{\mathrm{I}} = C^+ \wedge C^- \tag{10}$$

$$\mathrm{RF}_{\mathrm{II}} = C^+ \tag{11}$$

$$\tag{12}$$

The receptive fields of the detectors of type I and II are illustrated in Fig. S3.

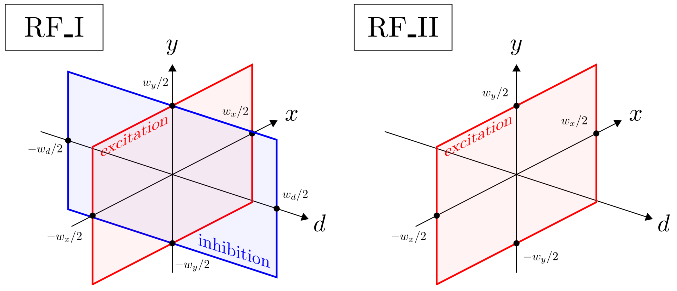

Figure S3. Receptive fields of two types of disparity detectors with (RF_{I}) and without inhibitory zone ($\mathrm{RF}_{\mathrm{II}}$).

S1.6 Mutual inhibition of disparity detectors

As the disparity detectors are not expected to address the problem of normalization, a fixed threshold $\theta_{\mathbf{d}}$ will result in one of two problems. If the threshold is low, disparity detectors are more likely to respond to false but similar targets, particularly at locations where the targets are large or rapidly moving (many coincidences), even in the presence of inhibiting evidence. In contrast, if the threshold is high, although the sensitivity to false disparities is reduced, the response to true disparities associated with small or slowly moving targets is also diminished (fewer coincidences). This suggests that $\theta_{\mathbf{d}}$ is a context-dependent and critical parameter. The proposed network addresses this problem by implementing the uniqueness constraint first described by.[2] Given a disparity detector which is spatially represented at the position of a false disparity, the correct disparity is located somewhere along the line of sight and is simultaneously represented by another neuron. Ideally, this neuron integrates more coinciding evidence, thus evoking a faster response. This response can then be recurrently fed as an inhibitory input into the neuron located at the false disparity in order to suppress its response. On a macro level, this concept results in winner-take-all synchronization among neurons on the same line of sight. In the proposed network, this mechanism is implemented with mutual inhibition, such that each disparity detector inhibits all the other neurons in its line of sight. Thus, Eq. 6 needs to be updated by subtracting an inhibitory current $I_{\mathbf{d}-}(t)$ accordingly:

$$\begin{cases} \tau_{\mathbf{d}} \dfrac{\mathrm{d}v_{\mathbf{d}}(t)}{\mathrm{d}t} = -v_{\mathbf{d}}(t) + I_{\mathbf{d}}(t) - I_{\mathbf{d}-}(t)\,, & v_{\mathbf{d}}(t) < \theta_{\mathbf{d}} \\ v_{\mathbf{d}}(t) = 0\,, & v_{\mathbf{d}}(t) \geq \theta_{\mathbf{d}} \vee v_{\mathbf{d}}(t) \leq 0 \end{cases} \tag{13}$$

whereby the inhibitory current is defined as

$$I_{\mathbf{d}-}(t) = w_{rec} \sum_{\mathbf{d} \in D^-} \sum_{n} \delta_{\mathbf{d}}(t - t_n) \tag{14}$$

and the inhibitory region D^- is defined by the two lines of sight (one from each retina):

$$D^- = \left\{ \mathbf{d} \in D \;\middle|\; (x_d - d_d = 2\bar{x}_L) \vee (x_d + d_d = 2\bar{x}_R) \right\} \tag{15}$$

If the recurrent synaptic weights are set such that $w_{rec} \geq \theta_{\mathbf{d}}$, then each spike that signals a correct disparity will completely reset the membrane potential of the other neurons in D^-. This is preferable as it prevents neurons at false disparities from gaining a head start when competing to signal the next correct disparity directly after a spike. In order to avoid a negative bias, a further condition is added to Eq. 13, that does not admit potentials below zero.

S1.7 Representation and coding of disparity

If a disparity detector is placed at each spatial location in $\mathbb{D}^3$, neighboring neurons have strongly overlapping receptive fields. A natural consequence is that disparity detectors also respond to targets which are at their preferred disparity, but distant from their preferred cyclopean position. This behavior corresponds exactly to real disparity-tuned complex cells, which are known to be position invariant. This means that such cells respond equally to any target within their receptive fields, regardless of its exact position. Thus, a homogeneously distributed population of disparity detectors in $\mathbb{D}^3$ produces redundant responses. Therefore, a broader spacing in the direction of the cyclopean coordinates (but not in the direction of disparity) is suitable. If the output of the network is considered, the entirety of all disparity spikes would correspond to a blurry dynamic disparity map, as disparity detectors are broadly tuned to cyclopean position. However, an accurate disparity map can be obtained by combining the output from coincidence and disparity detectors. Recall that coincidence detectors encode the entirety of all possible disparities (corresponding to true and false targets). Thus, if a disparity neuron spikes, the simultaneous responses of coincidence detectors that have an equal (or nearby) spatial representation in $\mathbb{D}^3$ reveal the correct and accurate disparities. Once again, the notion of events is useful to express the disparity response of the network. A *unipolar disparity event* $e_d^+ = (\mathbf{d}, t)$ represents a relative change in light intensity, that occurred at time t, at location $\mathbf{d}$, in $\mathbb{D}^3$. Similarly, a *unipolar coincidence event* $e_c^+ = (\mathbf{c}, t)$ represents coinciding evidence of a change in light intensity at location $\mathbf{c}$ in $\mathbb{D}^3$ at time t. The entirety of all coincidence and disparity events are defined by the sets $\mathcal{C}^+ = \{ e_c^+ \}$ and $\mathcal{D}^+ = \{ e_d^+ \}$ respectively. The final output of the network that is obtained from the combination of coincidence and disparity responses is then defined as the set of *filtered unipolar disparity events*:

$$\mathcal{O}^+ = \mathcal{C}^+ \cap \mathcal{D}^+ \tag{16}$$

The set $\mathcal{O}^+$ encodes spatially precise and unambiguous light intensity changes in disparity space. The network is extended by also considering the sign of intensity change, which is encoded using an additional event attribute, the polarity $s \in [-1, 1]$. In order to handle the polarity properly, a pair of coincidence detectors, one for each polarity, is used at each location in disparity space, similar to the simple ON and OFF cells in V1. Thus, the definition of coincidence events is modified accordingly: $e_c = (\mathbf{c}, s, t)$. Disparity detectors give equal weight to the evidence from both types of coincidence detectors. Thus, the polarity of the disparity detectors themselves is neutral. However, the filtered disparity events inherit the polarity of the coincidence events, which results in a set $\mathcal{O}$, that encodes spatially precise and unambiguous, polarized light intensity changes in disparity space:

$$\mathcal{O} = \left\{ e_c \in \mathcal{C} \;\middle|\; [e_c]^+ \in \mathcal{D}^+ \right\} \tag{17}$$

where $[]^+$ denotes a polarity rectification that transforms the polarity event $e_c = (\mathbf{c}, s, t)$ to a unipolar event $e_c = (\mathbf{c}, t)$.

S2 Natural disparity tuning curves

It is interesting to take a closer look at the behavior of disparity detectors when mutual inhibition is deactivated. This reveals the tuning functions and selectivity to false disparities of these detectors. A general problem in neuroscience that is associated with measuring tuning functions is that they depend on many aspects of the stimuli. In the past, for example, simple bars or sinusoidal gratings were often used to study the response of biological disparity neurons. In that case, obviously, the tuning functions of the disparity neurons show an unambiguous peak at their preferred disparity. From such an ideal tuning function it is often not clear how the cell responds to the more complex stimuli which occur in natural scenes. In an analogous manner, the tuning curves of the disparity neurons modeled in this work would correlate with the structure of the receptive field when tested with simple stimuli. In the case of the proposed disparity neurons, therefore, broad tuning to horizontal cyclopean position (position invariance) and fine tuning to disparity would be expected. This corresponds to the ideal definition of a disparity

detector. The following experiment examines the disparity tuning curves of the disparity neurons when stimulated by natural inputs. The method used for probing the tuning curves is detailed in the Methods section. The natural tuning curves which were obtained are shown in Fig. S4b for varying relative cyclopean positions and in Fig. S4c for varying relative disparities. Both tuning curves show the highest response at the true disparity $(\bar{x} = 0, \bar{d} = 0)$, while being broadly tuned to $\bar{x}$ and finely tuned to $\bar{d}$. These results indicate that the neurons are very well suited to be natural disparity detectors, as the response does not vary according to the position and shape of the stimulus. Interestingly, the tuning curve in Fig. S4c exhibits troughs on either side of the preferred disparity. This is an important characteristic of disparity detectors that suggests that they are not selective to false disparities within their receptive field. Similar characteristics can be observed in the tuning functions of real disparity-tuned cells. As a comparison, the predicted tuning curve from the disparity energy model of Ohzawa et al.[3]is shown, which also has broad position and narrow disparity selectivity.

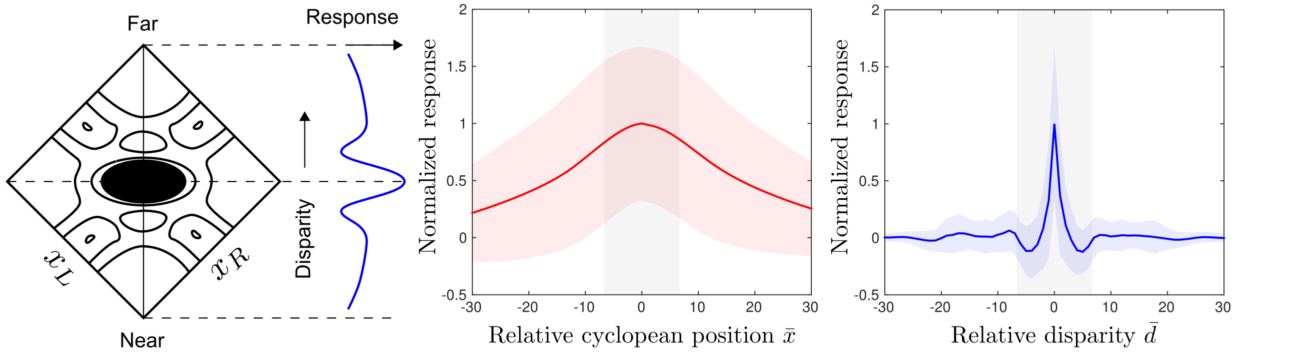

(a) (b) (c)

Figure S4. Tuning curves of natural disparity detectors. (a) Predicted tuning curve from the disparity energy model (adapted from Ohzawa et al.[3]). (b) Average neural response of type I disparity detector neurons for varying relative cyclopean position $\bar{x}$. (c) Average neural response for varying relative disparity $\bar{d}$. The solid line represents the mean, while the standard deviation is depicted by the shaded and colored area. The gray-shaded region indicates the width of the receptive field (here $\omega = 13$).

Probing of disparity tuning curves

The disparity tuning curves of the stereo network were probed according to the following procedure. When a true disparity occurs, the responses of neurons not tuned to the true disparity are recorded. More specifically, if a disparity event occurs at (x, y, d), the neural responses of interest are those which have preferred spatial locations distributed along the directions of x and d. The scene consisted of a face performing all kinds of movement, including translation and rotation. For each of the $600,000$ true disparity events, the membrane potentials of 60 neurons with the same cyclopean position but different disparity $d \in [d-30, d+30]$, and 60 neurons with the same disparity but different position $x \in [x-30; x+30]$ were sampled. Not just mutual inhibition, but also the threshold non-linearity of the disparity neurons was removed, in order to study the pure effect of coinciding evidence integration. Finally, all samples were shifted according to the neuron's spatial location in order to receive relative measurements that could be averaged and normalized.

S3 Spatiotemporal correlation mechanism of complex disparity detectors

Three different types of disparity detectors were proposed, which aim to approximate a sort of local correlation of spatiotemporal visual input in the form of spike events that encode temporal contrast changes. The proposed models do not account for normalized responses. Thus, it was suggested that the underlying mathematical computation is better described by the covariance rather than a normalized form of cross-correlation (e.g. NCC). In the following experiment, the responses of the three types of disparity detectors were recorded and compared to the result of the covariance computation. Fig. S5 illustrates the experimental procedure. The spiking visual input is represented in the form of *bipolar temporal images*, which are temporal images that also encode the sign of the intensity changes (polarity). To obtain a bipolar temporal image, a temporal image is multiplied one element at a time by the polarity of the last event at each spatial position. For this experiment, the images were cropped to match the receptive field size of the disparity neuron. Images were extracted in pairs from the left and right visual source at the locations of temporally coinciding events with epipolar spatial positions. For each such pair, regardless of whether it corresponded to a true or false match, the spike times were fed into a network. This network employed a single disparity detector and its response was recorded as the value of the membrane potential $v_{\mathbf{d}}$ (the threshold non-linearity was removed).

Simultaneously, the covariance of the image pair was computed as

$$\text{cov} = \sum_{\mathbf{p}} \left(\Sigma_L^+(\mathbf{p}) - \mu_{\Sigma_L^+}\right) \left(\Sigma_R^+(\mathbf{p}) - \mu_{\Sigma_R^+}\right) \tag{18}$$

where Σ_L^+ and Σ_R^+ are the bipolar temporal images and $\mu_{\Sigma_L^+}$ and $\mu_{\Sigma_R^+}$ are their associated mean values.

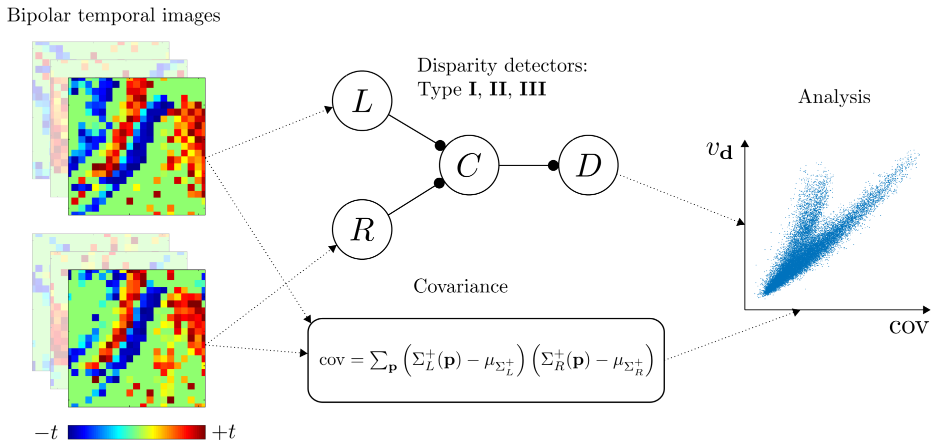

Figure S5. Schematic of the experimental procedure to investigate the underlying computation of the proposed disparity detectors.

S3.1 Type I and II disparity detectors

Type III disparity detectors use the direct inhibitory input from retinal cells to reduce the neuron's response at locations of non-coinciding interocular evidence. Previously, it was observed that activity in the plane of constant horizontal cyclopean position E_x can be used as an alternative cue when the spatiotemporal attributes of the stimuli do not comply. In order to corroborate this hypothesis, two further types of disparity detectors are examined. Type II is the simplest detector that only receives excitatory input from coincidence of constant disparity; thus, it constitutes the baseline behavior. Additionally, type I receives inhibitory input from coincidences within E_x. The experimental procedure was the same as before. This time, however, two DAVIS sensors with lower spatial resolution were used. The experiment was repeated for two different scenes, one of which was similar to the first scene of a moving face, and the other comprising two people passing by each other at different depths. The PCC was used as the statistical measure to determine the degree of correlation between the response of the disparity detectors and the covariance of temporal images. The values of the PCC for both types of detectors are graphed in relation to the receptive field width in Fig. S6. For both scenes, the response of type I neurons is more strongly related to the covariance of temporal images. This supports the idea that activity in E_x alludes to counter-evidence to spatiotemporal correlation. It can also be observed that the PCC always increases with the receptive field width. This result is expected, given that when large numbers of coincidence detectors are integrated, the highly non-linear thresholding effect is averaged out.

S4 Ground-truth evaluation method

When evaluating complex dynamic scenes, it is hard to model the ground truth, but it can be measured. One possibility would be to use a reference video-camera stereo system that provides accurate disparity or depth maps. Such a method was recently introduced by.[4] Another possibility is to use a more reliable 3D acquisition technique such as structured light. Here, a Microsoft Kinect is used to acquire 3D ground-truth data. In order to register 3D events with the ground truth which has been measured, the relative position of the Kinect with respect to the event-based camera stereo system needs to be precisely known. This can be obtained from the stereo calibration of the Kinect sensor and one of the event-based cameras. Again, matched events are triangulated and binned into 30 ms time slices. In so doing, the reconstructed 3D point clouds are sequentially compared to the ground-truth point clouds, which have been acquired and temporally synchronized (obtained from one frame of the Kinect running at 30 Hz). The 3D error $\varepsilon_\mathbf{X}$ for each 3D event, representing a point in the reconstructed point cloud, is computed as the minimum distance between any 3D point and the temporally coinciding ground truth.

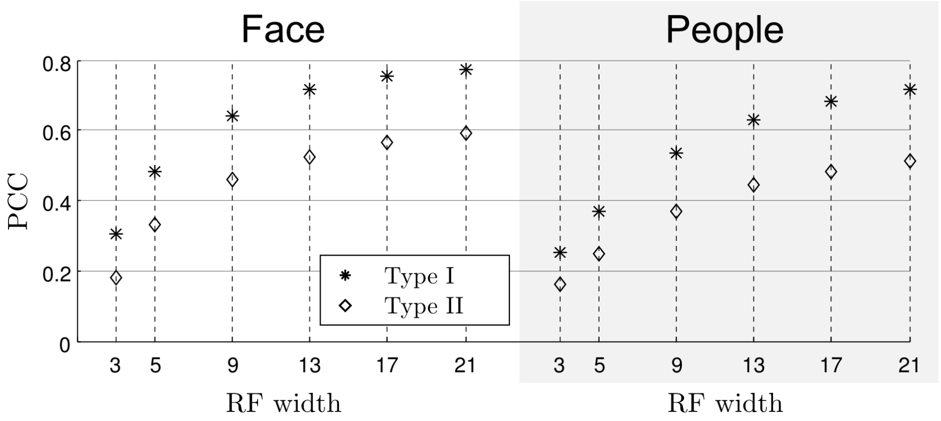

Figure S6. Evaluation of neural responses from two types of disparity detectors with (I) and without inhibition (II). The PCC reflects how well the neuron approximates the covariance of temporal images with respect to the width of the receptive field. Evidently, the type I disparity detector produces a closer approximation of the covariance of temporal images.

S4.1 Disparity error and matching performance metric

To assess the matching performance of the algorithm advocated in this paper, the error in disparity ε_d is calculated. This is a more meaningful metric than the 3D error $\varepsilon_{\mathbf{X}}$ because it does not vary for objects located at different depths. To determine the disparity error, the error in depth ε_Z needs to be measured. It is assumed that $\varepsilon_Z \approx \varepsilon_{\mathbf{X}}$ which is generally not correct. Theoretically, ε_Z should be measured as the distance from a reconstructed 3D point to its nearest neighbor in the ground-truth data, along a line of sight determined by the position of the 3D point and the center of the camera. This is the only way to ensure that the depth error is computed from physically corresponding points, rather than nearest neighbors which do not represent the same point. However, due to the poor resolution of the DAVIS sensor (which results in poor depth resolution) and an additional error introduced by the fact that the Kinect frames and 3D events are not perfectly temporarily synchronized, qualitative evaluations show that $\varepsilon_X \approx \varepsilon_Y \ll \varepsilon_Z$ and therefore the assumption $\varepsilon_Z \approx \varepsilon_{\mathbf{X}}$ holds. The disparity error can be computed from the depth error as follows:[5]

$$\varepsilon_d = \frac{bf}{Z^2}\varepsilon_Z \tag{19}$$

As previously discussed, the metric to evaluate the performance of stereo matching is based on the PCM, which can now be defined as:

$$PCM = \frac{1}{N_t}\sum_{i=1}^{N_t} h(e_{3D}^i) \quad \text{with} \quad h(e_{3D}) = \begin{cases} 1, & \varepsilon_d \leq 1 \\ 0, & \varepsilon_d > 1 \end{cases} \tag{20}$$

S5 Stereo matching performance

In the subsection which follows, the matching performance of the stereo network is assessed for three different dynamic scenes involving a moving face (1), two people moving in opposite directions (2), and a person performing martial arts (3). The results are qualitatively shown in Fig. S8, Fig. S9 and Fig. S10 respectively. The disparity error was computed as explained before and the histograms are individually shown in Fig. S7 for all scenes. The distribution of disparity errors fits very well a half-normal distribution:

$$f(x) = \frac{\sqrt{2}}{\sigma_d\sqrt{\pi}}\exp\left(-\frac{x^2}{2\sigma_d^2}\right) \qquad x > 0 \tag{21}$$

with a mean of $\mu_d = \sigma_d\sqrt{2}/\sqrt{\pi}$. Generally, it can be observed that the stereo network performs equally well for all scenes. Scene (2) yields the best results, while scenes (1) and (3) yield a slightly higher percentage of incorrect matches. This is likely to be due to the fact that scenes (1) and (3) both contain motion that is not fronto-parallel, whereas in scene (2), the two people are walking perfectly parallel to the baseline. The relatively constant and low disparity error throughout all the scenes suggests

that the matching performance is very robust and largely independent of how fast the objects move (illustrated by the event rate). In order to quantitatively measure performance, the percentage of true matches is computed, whereby a match is considered true if the associated 3D event had a disparity error of less than one pixel. This corresponds to the standard PCM of $\delta = 1.0$. Furthermore, the PCMs of $\delta = 2.0$ and $\delta = 3.0$ have been determined. These measures were found to be very high, indicating that although the majority of falsely matched targets are inaccurate, they are roughly correct. A summary of the results is listed in Tab. S1. Tab. S2 lists the scene statistics and the averaged results. All scenes contained a more or less equivalent number of input events, n_l and n_r, from the left and right sensors respectively. The number of coincidences n_c is similar for all scenes, which suggests that the correspondence problem is equally complex. Finally, the number of disparity events n_d matches the number of input events very well, which is desirable (unity gain). The average disparity error for the scene is denoted by $\bar{\varepsilon}_d$ and the depth error by $\bar{\varepsilon}_Z$ accordingly. It is clearly visible that $\bar{\varepsilon}_Z$ depends on the proximity of the scene objects. In scene (2), for example, the second person was located very far away (>4m) from the cameras. Conversely, the average disparity error $\bar{\varepsilon}_d$ is independent of proximity and remains constant for all scenes.

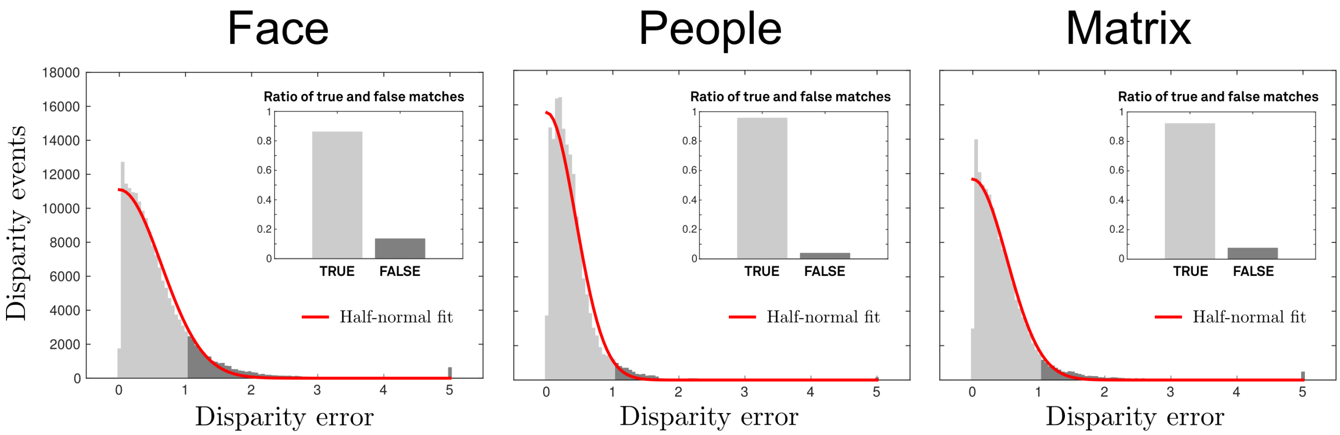

Figure S7. Disparity error histograms for all three scenes. Events corresponding to true matches (disparity error <1 pixel) are shaded light gray. All disparity errors that are greater than 5 pixels are contained in the last bin. A half-normal distribution was fitted to each of the histograms (red curve). The insets show the ratio of true and false matches. The "people" scene shows the best observable performance, evidenced by the ratio of true and false matches and the narrowness of the distribution of disparity errors.

Table S1. Summary of results.

Scene	PCM ($\delta = 1.0$)	PCM ($\delta = 2.0$)	PCM ($\delta = 3.0$)
Face	86.3%	97.7%	99.2%
People	95.9%	99.6%	99.9%
Matrix	92.3%	98.7%	99.5%
**Average**	**91.5%**	**98.7%**	**99.5%**

Table S2. Scene statistics and experimental results.

	Scene statistics					Averaged results				Fit	
Scene	T	n_l	n_r	n_c	n_d	$\bar{\varepsilon}_d$	s.d.	$\bar{\varepsilon}_Z$	s.d.	μ_d	σ_d
(1) Face	4.5	685k	518k	3'538k	699k	**0.64**	**2.04**	18.82	24.13	**0.50**	**0.63**
(2) People	4	666k	611k	3'250k	766k	**0.37**	**0.42**	85.77	110.08	**0.35**	**0.44**
(3) Matrix	3.0	557k	430k	3'174k	582k	**0.53**	**1.91**	32.57	32.38	**0.41**	**0.51**

Video S1. Video showing the results of scene (2). The different views show the input (left), the network activity (middle) and 3D events (right). *Left view:* Combined frames of accumulated input from the left (green) and right (purple) sensors. *Middle view:* Network output shown as disparity maps generated from accumulated filtered disparity events. As explained in the text, filtered disparity events occur immediately after a coincidence event with the same disparity space coordinates. These events are considered to be the final output of the network. *Right view:* 3D events generated by triangulating disparity events, whose z coordinate is color-coded to improve 3D visibility.

References

1. Gerstner, W. & Kistler, W. M. *Spiking Neuron Models: Single Neurons, Populations, Plasticity* (Cambridge University Press, 2002).
2. Marr, D. & Poggio, T. Cooperative computation of stereo disparity. *Science* **194**, 283–287 (1976).
3. Ohzawa, I. Mechanisms of stereoscopic vision: the disparity energy model. *Current Opinion in Neurobiology* **8**, 509–515 (1998).
4. Kogler, J., Eibensteiner, F., Humenberger, M., Gelautz, M. & Scharinger, J. Ground Truth Evaluation for Event-Based Silicon Retina Stereo Data. In *2013 IEEE Conference on Computer Vision and Pattern Recognition Workshops (CVPRW)*, 649–656 (2013).
5. Gallup, D., Frahm, J.-M., Mordohai, P. & Pollefeys, M. Variable baseline/resolution stereo. In *IEEE Conference on Computer Vision and Pattern Recognition, 2008. CVPR 2008*, 1–8 (2008).

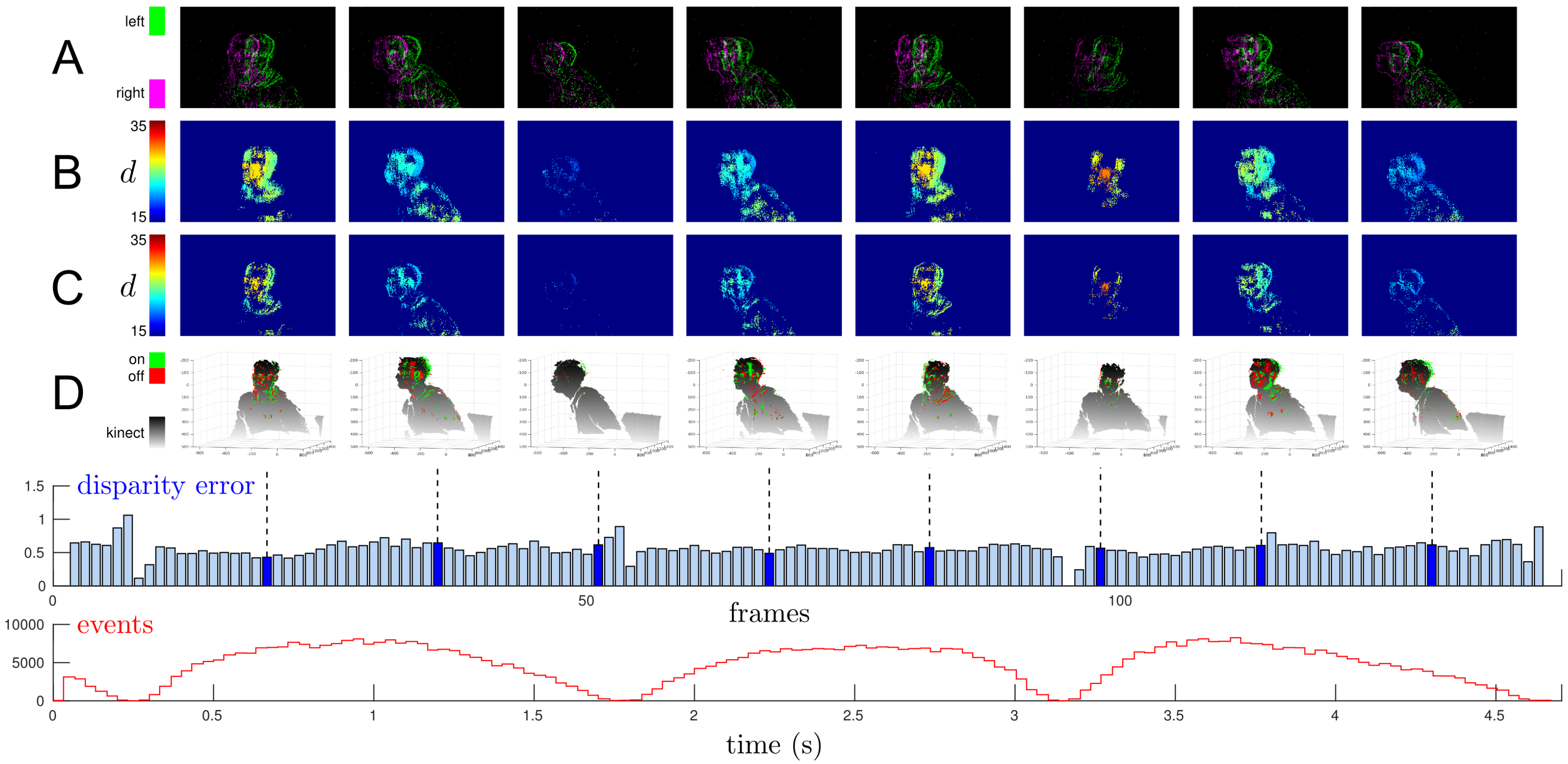

Figure S8. Qualitative and quantitative results of scene (1). This scene comprises a face that first rotates to the right (from its point of view) followed by a rotation to the left while moving towards the cameras. Finally, it rotates again rightwards and recedes. The scene lasts 5 seconds. On the bottom, the disparity error and input event rate are plotted over time. The disparity error remains largely constant even when the input event rate strongly varies. The series of images show the input (A), the network activity (B,C) and 3D events (D) at the frames/time indicated. (A) Combined frames of accumulated input from the left (green) and right (purple) sensors. (B) Disparity maps generated from accumulated unfiltered disparity events. (C) Disparity maps generated from accumulated filtered disparity events. As explained in the text, filtered disparity events occur immediately after a coincidence event with the same disparity space coordinates. These events are considered to be the final output of the network. (D) 3D events generated by triangulating disparity events, whose polarity is color-coded in green (ON) and red (OFF). The ground-truth point cloud which was obtained from the Kinect is also shown (gray).

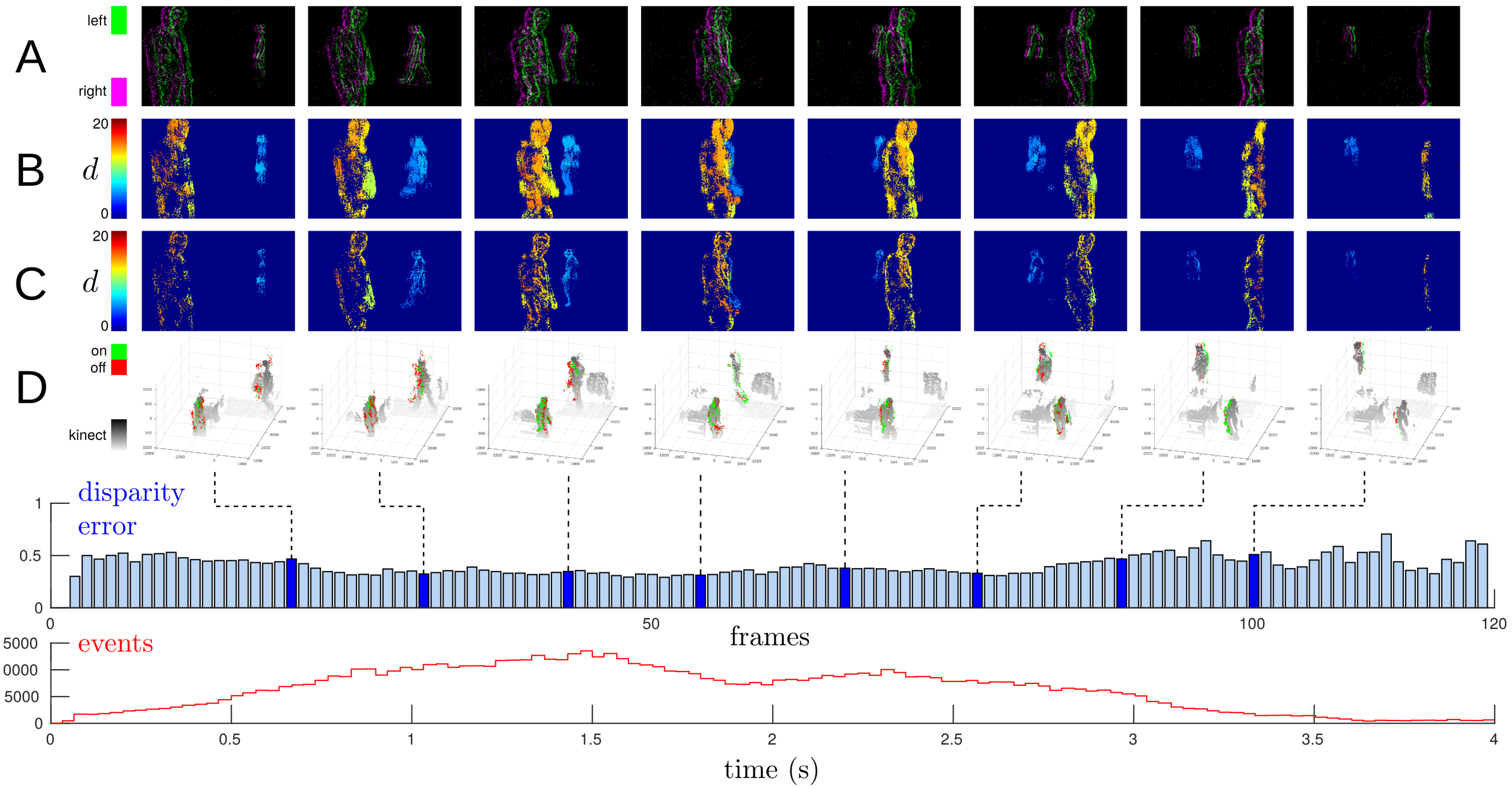

Figure S9. Qualitative and quantitative results of scene (2). This scene comprises two people that are walking in opposite directions at different depths. The scene lasts 4 seconds. At the bottom, the disparity error and input event rate are plotted over time. The scene features large disparity gradients (at the point when the people cross each other) and is therefore considered a difficult matching problem. As can be seen from the disparity maps located in the middle, the network unambiguously detects both people. This is explained by the strong motion cues that are present in this particular scenario. The series of images show the input (A), the network activity (B,C) and 3D events (D) at the frames/time indicated. (A) Combined frames of accumulated input from the left (green) and right (purple) sensors. (B) Disparity maps generated from accumulated unfiltered disparity events. (C) Disparity maps generated from accumulated filtered disparity events. As explained in the text, filtered disparity events occur immediately after a coincidence event with the same disparity space coordinates. These events are considered to be the final output of the network. (D) 3D events generated by triangulating disparity events, whose polarity is color-coded in green (ON) and red (OFF). The ground-truth point cloud which was obtained from the Kinect is also shown (gray).

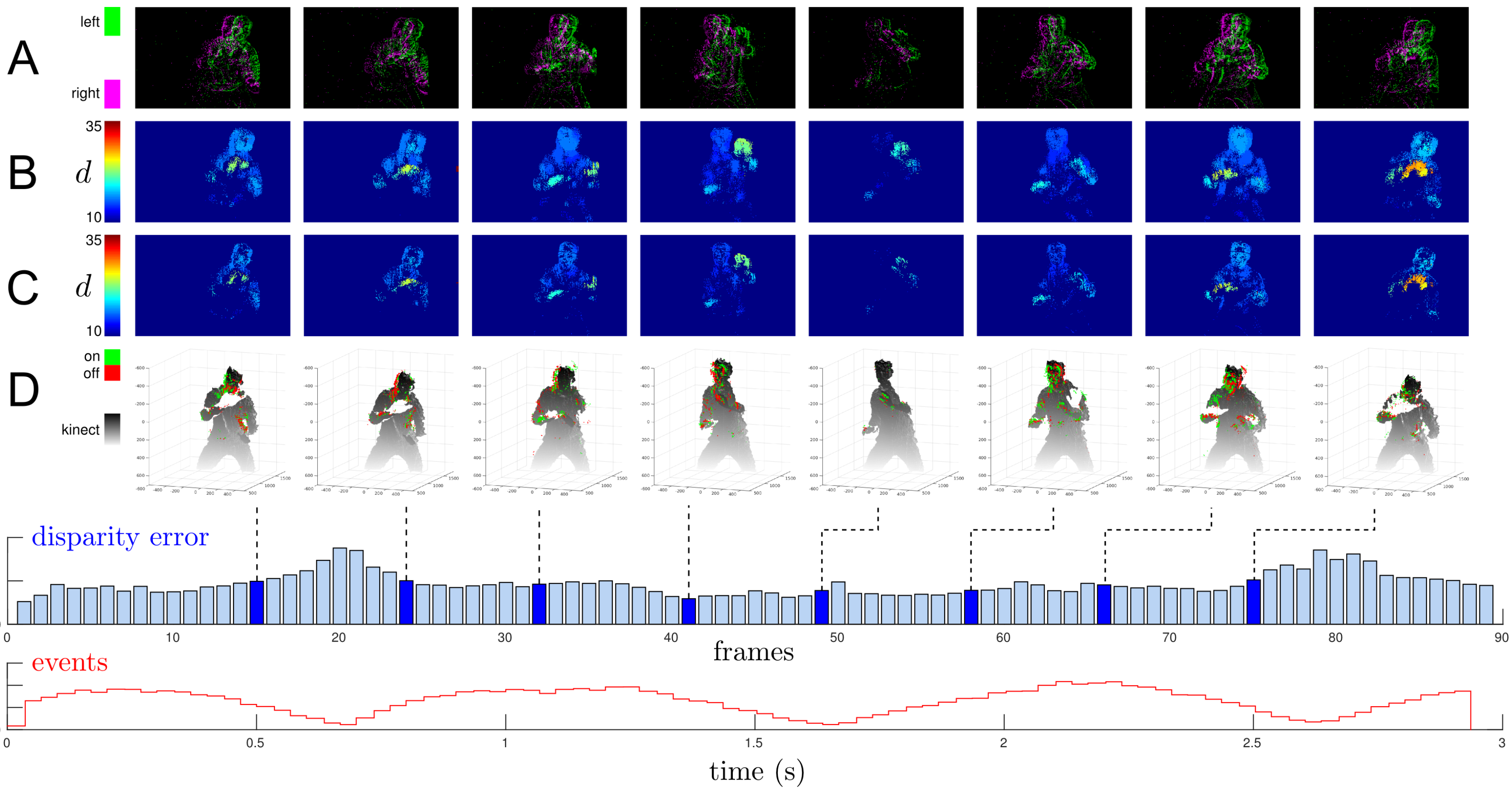

Figure S10. Qualitative and quantitative results of scene (3). This scene comprises a person performing martial arts. The scene lasts 3 seconds. At the bottom, the disparity error and input event rate are plotted over time. This scene combines large disparity gradients and various motion cues. Although the hands are partially moving forwards and backwards, the network is still capable of solving the correspondence problem. Here, it can be observed that the disparity error slightly increases when the person moves more slowly (decreased event rate). This is because there are no motion cues at these points, which are required to correctly match large disparity gradients. The series of images show the input (A), the network activity (B,C) and 3D events (D) at the frames/time indicated. (A) Combined frames of accumulated input from the left (green) and right (purple) sensors. (B) Disparity maps generated from accumulated unfiltered disparity events. (C) Disparity maps generated from accumulated filtered disparity events. As explained in the text, filtered disparity events occur immediately after a coincidence event with the same disparity space coordinates. These events are considered to be the final output of the network. (D) 3D events generated by triangulating disparity events, whose polarity is color-coded in green (ON) and red (OFF). The ground-truth point cloud which was obtained from the Kinect is also shown (gray).
